# Expression of Cancer Stem Cell Marker CD44 and Its Polymorphisms in Patients with Chronic Gastritis, Precancerous Gastric Lesion, and Gastric Cancer: A Cross-Sectional Multicenter Study in Thailand

**DOI:** 10.1155/2017/4384823

**Published:** 2017-12-27

**Authors:** Taweesak Tongtawee, Wareeporn Wattanawongdon, Theeraya Simawaranon, Soraya Kaewpitoon, Sivamate Kaengpenkae, Nichaphat Jintabanditwong, Pakorn Tangjanyatham, Warisara Ratchapol, Kokiet Kangwantas, Chavaboon Dechsukhum, Wilairat Leeanansaksiri, Natthawut Kaewpitoon, Likit Matrakool, Sukij Panpimanmas

**Affiliations:** ^1^School of Surgery, Institute of Medicine, Suranaree University of Technology, Nakhon Ratchasima 30000, Thailand; ^2^Suranaree University of Technology Hospital, Nakhon Ratchasima 30000, Thailand; ^3^School of Family Medicine, Institute of Medicine, Suranaree University of Technology, Nakhon Ratchasima 30000, Thailand; ^4^Department of Surgery, Surin Hospital, Surin 32000, Thailand; ^5^Buriram Hospital, Buriram 31000, Thailand; ^6^School of Oncology, Institute of Medicine, Suranaree University of Technology, Nakhon Ratchasima 30000, Thailand; ^7^School of Pathology, Institute of Medicine, Suranaree University of Technology, Nakhon Ratchasima 30000, Thailand; ^8^School of Preclinic, Institute of Science, Suranaree University of Technology, Nakhon Ratchasima 30000, Thailand; ^9^Faculty of Public Health, Vongchavalitkul University, Nakhon Ratchasima 30000, Thailand

## Abstract

Here we investigated CD44 protein expression and its polymorphisms in patients with chronic gastritis, precancerous gastric lesions, and gastric cancer; and we evaluated our result with the risk of CD44 protein expression and clinicopathological characteristics. Our results obtained by analyzing 162 gastric cancer patients, 125 chronic gastritis, and 165 precancerous gastric lesions from three study centers in Thailand showed that CD44 expression was significantly higher in patients with precancerous gastric lesions and gastric cancer while patients with chronic gastritis were negative for CD44 staining (*p* = 0.036). We further observed the significant association of variant genotype; gastric cancer patients carrying AG or GG of CD44 rs187116 had more increased risk of CD44 expression than wild-type (WT) carriers (AG: odds ratio (OR) = 5.67; 95% CI = 1.57–7.23; *p* = 0.024 and GG: OR = 8.32; 95% CI = 2.94–11.42; *p* = 0.016), but no significant difference in the risk of CD44 expression due to polymorphism in patients with precancerous gastric lesions. Our results suggested that CD44 expression could be used as a marker for the prediction of gastric cancer development, particularly in patients with precancerous gastric lesions carrying AG or GG, who were selected to surveillance follow-up for gastric cancer prevention.

## 1. Introduction

Gastric cancer is the fifth most common human cancer and the third leading cause of cancer-related death worldwide [1]. In Thailand, the incidence of gastric cancer is 5–7 in 100,000 people and it occurs predominantly in people aged between 60 and 70 years [2]. Currently, it is well established that* H. pylori* infection is the main etiological risk factor for gastric cancer, which progresses through a multiple processes, developing from gastritis, gastric atrophy, intestinal metaplasia, dysplasia, and finally to carcinoma [3]. Other factors, such as diets high in salt, a low intake of fruits and vegetables, tobacco smoking, and family history of stomach cancer, may therefore synergistically trigger gastric carcinogenesis. Moreover, the etiologic factors include multiple genetic and epigenetic alterations are implicated in the multiprocess of the development and spread of gastric cancer [4]. The prognosis of advanced gastric cancer remains poor while early gastric cancer is associated with excellent long-term survival [5]. Therefore, efforts to identify marker that could be used to detect early stage of gastric cancer or premalignant gastric lesions are of great clinical importance.

Cancer stem cells (CSCs) are a newly proposed theory for tumor development and form the basis for tumor proliferation and metastasis. CSCs are a subgroup of tumor cells that have the capacity of self-renewal and multilineage differentiation [6]. It has been reported to be involved in the carcinogenesis of a variety of malignancies [7]. Several studies have shown that CSCs could play a crucial role in the initiation and progression of gastric cancer, as in other gastrointestinal tumors [8–11]. Cluster of differentiation 44 (CD44) is a cell surface glycoprotein that plays an important role in many cellular processes, including regulation of cell division, adhesion, migration, and survival through the binding of its major ligand, hyaluronic acid [12]. CD44 has been identified as one of the cell surface markers associated with cancer stem-like cells in various solid tumors [13–15]. CD44 expression has also been reported in gastric cancer and has been suggested as a useful predictive marker in patients with gastric cancer [16–18].

Single nucleotide polymorphisms (SNPs) are the most common type of DNA sequence variation, and the expression of certain genes may be affected by their genetic variations. The effect of CD44 polymorphisms on human cancer susceptibility and clinical outcome has been described in various human cancer studies [19–21]. There are many studies to test the effects of several CD44 gene polymorphisms on cancer risk [22–24]. Among them, the rs187116 A>G polymorphism, located in intron 1 of CD44 gene, is the most frequently studied [19, 25]. Moreover, CD44 rs187116 genotype has been suggested for use as identifying and predicting the clinical outcome of gastric cancer patients [19, 23]. Despite there being many studies of the expression of CD44 in gastric cancer worldwide, it is unknown which CSCs marker could be more effective to predict gastric cancer development. Furthermore, there is lack of information about the corresponding of CD44 expression and the progression of gastric cancer in precancerous gastric lesions. Based on the implications of these previous studies, we hypothesized that CD44 expression may be related to the development of gastric cancer and genetic polymorphisms in CD44 may affect their expression; it may be associated with risk of developing cancer. Therefore, this study aimed to investigate the expression of CD44 protein and its polymorphisms in patients with chronic gastritis, precancerous gastric lesions, and gastric cancer; we evaluated our results with the risk of CD44 protein expression and clinicopathological characteristics of gastric cancer such as location of tumor, tumor size, histologic type, lymphatic invasion, vascular invasion, pathological T stage, pathological TNM stage, residual tumor, CEA, and 5-year survival. The findings may help to select patients at high risk for tumor development who might benefit from surveillance follow-up for gastric cancer prevention.

## 2. Materials and Methods

### 2.1. Patients and Specimens

Samples were obtained from patients with chronic gastritis, precancerous gastric lesions, and gastric cancer who underwent esophagogastroduodenoscopy (EGD) or surgical resection at Suranaree University of Technology Hospital, Buriram Hospital Medical Center, and Surin Hospital Medical Center from Northeast Thailand during January 2011 to December 2015. Written informed consent was obtained from all patients, and the study protocol was approved by the Ethics Committee for Research Involving Human Subjects, Suranaree University of Technology (EC-59-45 and EC 16-2560). The methods were carried out in accordance with good clinical practice and the guidelines of the Declaration of Helsinki.

### 2.2. Biopsy Specimens

The esophagogastroduodenoscopy (EGD) procedures were performed using an upper GI video endoscope (Olympus EVIS EXERA III, CV-190). The whole stomach was examined first with conventional endoscopy and then biopsies were performed by using “Site Specific Biopsy” technique [26]. Gastric tissue specimens for histological analysis were sent to the pathologist. The hematoxylin and eosin stain and Giemsa stain were used for identification of* H. pylori*. During histological examination, the presence of chronic gastritis, gastric atrophy, and intestinal metaplasia was assessed and graded by using 5 pathologists.

### 2.3. DNA Extraction

Genomic DNA was extracted from formalin-fixed, paraffin-embedded (FFPE) tissue of 300 gastric patients by using the QIAamp DNA FFPE tissue kit (Qiagen, Duesseldorf, Germany) according to the manufacturer's instructions. Briefly, the paraffin-embedded tissues were deparaffinized in xylene, hydrated in 100% ethanol, and digested by lysis buffer and proteinase K. Genomic DNA was purified from the tissue lysate using the QIAamp spin column and eluted. The isolated nucleic acid concentration and purity were determined in a DS-11+ spectrophotometer (Denovix, Wilmington, Delaware, USA) and stored at −20°C.

### 2.4. CD44 Genotyping

In order to characterize CD44 rs187116 (A>G) gene polymorphism, predesigned Custom TaqMan SNP Genotyping Assay was performed. They were selected according to the SNP database of the National Center for Biotechnology Information. The genotype of CD44 polymorphisms was determined by TaqMan allelic discrimination using predesigned Custom TaqMan SNP Genotyping Assay by real-time PCR. Forward and reverse primers were used along with wild-type probe VIC and probes FAM used for variant allele. Primers and probes were supplied by Applied Biosystems. Real-time PCR was performed using LightCycler® 480 II instrument (Roche diagnostics, Neuilly sur Seine, France) according to the manufacturer's instructions. Briefly, the PCR conditions were as follows: 95°C for 10 min, 55 cycles of 95°C for 15 s, and 60°C for 1 min. The success rate of genotyping was more than 94%. Negative controls and duplicate samples were used to check the accuracy of genotyping and initially analyzed with LightCycler 480 Software 1.5 (Roche diagnostics, Neuilly sur Seine, France).

### 2.5. Immunohistochemistry

Immunohistochemical staining was performed by using 4 *μ*m sections of formalin-fixed, paraffin-embedded tissue samples. The avidin-biotin complex method (ABC; Thermo Fisher, Illinois, USA) was used for immunohistochemical detection of CD44. Briefly, sections were deparaffinized in xylene and rehydrated in a graded alcohol. After washing in distilled water, the sections were microwaved in 10 mm citrate buffer (pH 6.0) for antigen retrieval. The tissue sections were then incubated in 1.5% normal blocking serum, followed by incubated with monoclonal mouse HCAM antibody (dilution 1 : 100, clone DF1485; Santa Cruz Biotechnology, Santa Cruz, CA) overnight at 4°C. Subsequently, the tissue sections were rinsed and incubated with the biotinylated goat anti-mouse secondary antibody (1 *μ*g/ml) for 30 min at room temperature, followed by incubation with HRP-conjugated avidin-biotin-complex (Thermo Fisher, Illinois, USA) for 30 min at room temperature. The specific bindings of antibodies within the tissue sections were visualized with the aminoethyl carbazole substrate solution (Life technologies Corporation, Carlsbad, California, USA), followed by counterstaining with Mayer's hematoxylin, dehydrating, and coverslipping.

### 2.6. Immunohistochemistry Evaluation

The immunohistochemical staining results were evaluated by two independent pathologists, who were blinded to the clinicopathological details of the patients. The assessment of CD44 stainings (score) was based on the percentage of positive cells: lack of staining was scored as negative and 1–50% were classified as “1+”, >10 and ≤50% as “2+” and >50% as “3+”. The cases classified as 0 were considered negative, whereas 1+, 2+, and 3+ were established as positive cases.

### 2.7. Statistical Analysis

All statistical analyses were carried out using the SPSS used for Windows (version 20.0; SPSS, Chicago, IL, USA). Comparison between the groups was done by using ANOVA for patient's demographic data. The statistical significance of any associations between CD44 polymorphism and CD44 protein immunohistochemical staining and clinicopathological factors of gastric cancer such as location of tumor, tumor size, histologic type, lymphatic invasion, vascular invasion, pathological T stage, pathological TNM stage, residual tumor, CEA, and 5-year survival was evaluated using the *χ*2 and Fisher's tests. To assess prognostic index, we first performed by using univariate Cox regression model analysis. Significant parameters from the univariate analysis were then assessed in the final by using multivariate analysis using Cox proportional hazards regression modeling with step-wise forward selection, *p* < 0.05 considered as statistically significant.

## 3. Results

### 3.1. Patient Characteristics

The patient characteristics are summarized in Table 1. In total, 125 chronic gastritis cases (35.5% males and 64.5% females), 113 precancerous gastric lesion cases (39.3% males and 60.7% females), and 162 gastric cancer cases (72.5% males and 27.5% females) were recruited. The median age of chronic, precancerous, and gastric cancer was 55.03 ± 12.92, 54.32 ± 15.83, and 61.38 ± 12.39 years, respectively. There were no significant differences between chronic gastritis and precancerous lesions for gender, age, or underlying conditions (Table 1).

### 3.2. Genotype Patterns of CD44 Polymorphism in Gastric Mucosal Pathology

**Figure 1:**
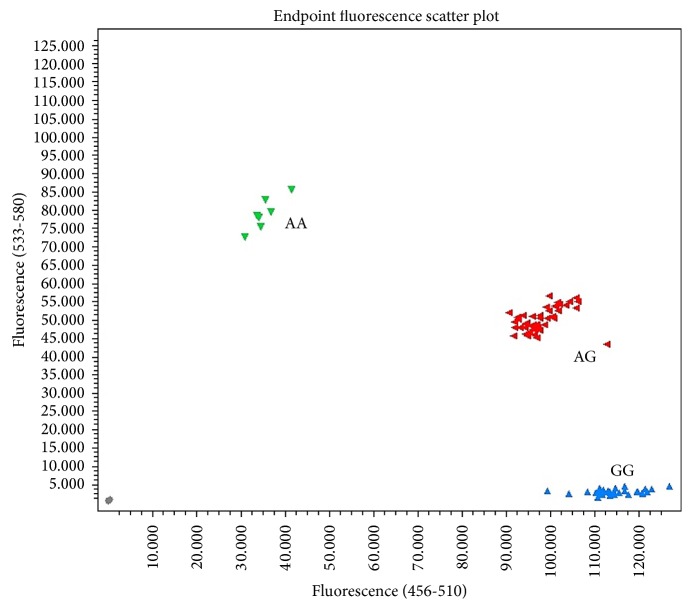
The patterns of CD44 polymorphism from real-time PCR.

The patterns of CD44 polymorphism are shown in Figure 1. The frequency of genotypes AA, AG, and GG in precancerous gastric lesion patients were 17.9%, 39.3%, and 42.8%, respectively, while that in the gastric cancer were 2.5%, 42.5%, and 55%, respectively. The AA homozygous showed significant difference between chronic, precancerous gastric lesion,s and gastric cancer groups (*p* = 0.024) while the distribution of variant genotypes (AG and GG) was not statistically significant difference between three groups (Table 1).

### 3.3. Immunohistochemical Analysis for CD44 Expression in Gastric Mucosal Pathology

**Figure 2:**
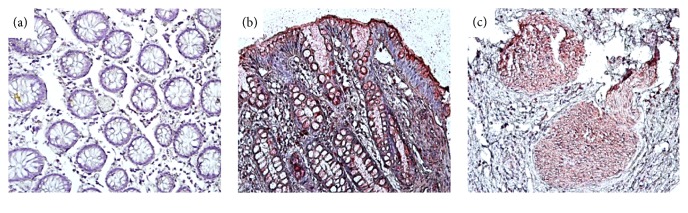
Representative photomicrograph of immunohistochemistry CD44 expression in gastritis tissue. CD44 staining in chronic gastritis (a), precancerous gastric lesions (b), and gastric cancer (c) (magnification, ×200).

We analyzed the expression levels of the CD44 protein using immunohistochemistry of 300 patients with chronic gastritis, precancerous gastric lesions, and gastric cancer. CD44 was stained mainly in the membrane and cytoplasm of gastric cells (Figure 2). The expression of CD44 protein was present in patients with precancerous gastric lesions (21.4%) and gastric cancer (65%) but no patients with chronic gastritis were positive for CD44 staining. There are also significant differences in CD44 expression between three groups (*p* = 0.036) (Table 1).

### 3.4. Association between CD44 Genotypes and Protein Expression in Gastric Mucosal Pathology

In order to assess whether CD44 protein expression was due to alteration in CD44 genotypes, we further analyzed the association of genotype patterns and protein expression of CD44 in patients with chronic gastritis, precancerous gastric lesions and gastric cancer. In gastric cancer, 69 cases of positive CD44 protein expression carried AG genotype (35.89%) and 89 cases carried GG genotype (43.58%) whereas 4 cases carried AA genotype (2.56%). There were associations between CD44 genotype and positive of CD44 staining, AG and GG genotype showed significant associated with risk of positive for CD44 staining (*p* = 0.024 and *p* = 0.016, resp.). The OR of carrying AG and GG genotype in gastric cancer patient groups were 5.67 (95% CI = 1.54 to 7.23) and 8.32 (95% CI = 2.94 to 11.41) by using univariate Cox regression model analysis and 3.28 (95% CI = 2.47 to 5.63) and 4.14 (95% CI = 1.84 to 7.32), respectively, by using multivariate Cox proportional hazards regression model analysis. In contrast, the distribution of three genotypes and CD44 protein expression in chronic gastritis and precancerous gastric lesions were not statistically significant (Table 2).

### 3.5. Prognostic Role of CD44 Protein Expression and Clinicopathological Characteristics of Gastric Cancer

All tissue slides were re-reviewed by five pathologists. Positive expression rates were 65% for gastric cancer tissues. Clinicopathological relevance of background factors in gastric cancer patients is summarized in Tables 3 and 4. Location of tumor, tumor size, histologic type, lymphatic invasion, vascular invasion, pathological T stage, pathological TNM stage, residual tumor, and 5-year survival were significantly associated with risk of positive for CD44 staining (Table 3). Then multivariate Cox proportional hazard regression model was used to adjust incorporating all of the relevant factors, such as age, gender, underlying disease, and family history of gastric cancer. The results found that location of tumor (lower) [*p* = 0.018, OR = 3.82 (1.31–5.84)], tumor size (≥70 mm) [*p* = 0.023, OR = 5.24 (3.28–8.18)], undifferentiated histologic type [*p* = 0.037, OR = 4.19 (4.18–10.29)], absent lymphatic invasion [*p* = 0.018, OR = 4.12 (1.08–6.34)], T3 and T4 pathological stage [*p* = 0.041, OR = 2.09 (1.02–3.07)], high pathological stage (stages III and IV) [*p* = 0.026, OR = 3.46 (1.82–5.24) and *p* = 0.019, OR = 4.33 (3.47–8.89)], residual tumors [*p* = 0.022, OR = 3.29 (1.87–4.39)], and 5-year survival rate [*p* = 0.038, OR = 2.26 (2.01–4.89)] were all found to be significantly related to CD44 protein expression (Table 4). On the other hand, no significant differences in any clinicopathological characteristics were observed between CD44 protein-positive and negative groups. During the study period, 13 (10.31%) patients were lost to follow-up and 45 (30.20%) patients died. Of these, 41 (91.11%) patients died from recurrent gastric cancer and 4 (8.88%) patients died from another disease without recurrence of gastric cancer.

## 4. Discussion

According to CSC theory, CSCs can drive tumorigenic processes including cancer initiation, progression, metastasis, and disease recurrence [27]. Stem cells become cancerous after having acquired genetic mutations and seem to be responsible of recurrence and metastasis [28]. CD44 has been suggested to represent an important prognostic marker for various cancer types including gastric cancer with its elevated expression [16, 20, 22, 29, 30]. Several studies have demonstrated that overexpression of CD44 protein was associated with poor prognosis in colorectal carcinoma [30], breast cancer [22], and gastric cancer [16]. Based on the above, it is reasonable to predict that changes in the expression of CD44 will play an important role in the development and progression of gastric cancer. In addition, these CSCs are used as a marker for identifying and predicting gastric cancer development; they should be validated in several populations because of racial differences. This is the first report to evaluate CD44 protein expression and polymorphism in Thai gastritis patients. Our results obtained by analyzing 162 gastric cancer patients and 125 chronic gastritis and 165 precancerous gastric lesions from three study centers showed that CD44 protein staining was positive in patients with precancerous (21.4%) and gastric cancer (65%) and negative with chronic gastritis. There was significant difference in CD44 protein expression among groups of patients (*p* = 0.036). This result indicated the association between CD44 protein expression and development of pathological changes (Table 1). It might be suggested that CD44 expression could be used as a marker for the prediction of gastric cancer development, especially in patients with premalignant gastric lesions carrying AG or GG, who was selected to surveillance follow-up for gastric cancer prevention. Consistently, Dammrich et al. [31] reported that CD44 (v6 isomer) was expressed in the chronic atrophic gastritis and intestinal metaplasia with dysplastic change which are precancerous lesions of gastric carcinoma. Thus, the expression of stem cell markers can represent the epigenetic changes supporting the role of stem cells in gastric cancer. This study could therefore evaluate the association between CD44 protein expression and CD44 polymorphism and compare the risk of CD44 protein expression in precancerous gastric lesions and gastric cancer.

Several studies have suggested the potential importance of CD44 polymorphisms as a risk factor and poor prognostic marker in various cancers, including gastric cancer [19, 21, 29, 32]. Recently, a number of studies have conducted the association between CD44 rs187116 polymorphisms and risk of cancer; however, the results were conflicting. In this study, AA homozygous genotype showed a significant difference between chronic gastritis and both precancerous gastric lesions and gastric cancer (*p* = 0.024). This result indicated that patients with chronic or precancerous carried more AA homozygous genotype than gastric cancer patients. This might suggest that polymorphism contribute to the enhanced carcinogenesis as compared with wild-type. Particularly, the AG and GG genotypes of gastric cancer patients were found to increase by 17 and 22 times, respectively (Table 1). These findings correspond to the study of Suenaga et al. [23]; they reported that the most frequently genotypes in Japanese gastric cancer patients are AG and GG genotypes. Moreover, we observed that the significant association of variant genotype AG or GG of CD44 rs187116 had a higher risk of CD44 protein expression in gastric cancer compared with those with the WT genotype (AA). Although the G allele appeared to be associated with the risk of CD44 expression in gastric cancer, however no statistically significant was observed in precancerous gastric lesions; the results might reflect statistical fluctuation due to the small number of patients in each group. Therefore, the association between CD44 rs187116 polymorphism and the expression of CD44 protein in gastritis patients is inconclusive from the present study. Another explanation for this observation is that the CD44 protein expression may not result from CD44 mutation of rs187116 but mutations in genes that regulate carcinogenesis could result in uncontrolled CD44 expression and gastric cancer. In this study, CD44 rs187116 evaluated the effect of CD44 gene. Therefore, other variants should be evaluated for further study of gastric cancer carcinogenesis. Although this study was the multicenter providing subject from the three hospital centers, however, a large number of patients are needed. Moreover, selection bias was present in this study, mostly gastric cancer tissues showing locally advance and advanced gastric cancer, because specific amounts of gastric cancer tissues were collected from surgical resection from three medical centers.

## 5. Conclusions

To date no reliable CSCs marker is found to predict gastric cancer development. Our study indicate that expression of CD44 could be used as a marker for the prediction of gastric cancer development, especially in patients with precancerous gastric lesions, who were selected to surveillance follow-up for gastric cancer prevention. Larger multicenter studies are needed to test this hypothesis.

## Figures and Tables

**Table 1:** Patient's demographics data among gastric mucosal pathology.

Patient's demographics data	Gastric mucosal pathology			*p* value
	Chronic gastritis (*n* = 125)	Precancerous (*n* = 113)	Gastric cancer (*n* = 162)	
Age (year ± SD)	55.03 ± 12.92	54.32 ± 15.83	61.38 ± 12.39	0.169
Sex (male (%))	35.5	39.3	72.5	0.057
Underlying condition (%)				
(i) HT	9.67	7.14	10.25	0.861
(ii) DM	12.90	10.71	12.82	0.732
(iii) Hyperlipidemia	6.45	3.57	5.12	0.463
(iv) Smoking	16.12	14.28	15.38	0.548
(v) Alcohol	12.90	7.14	15.38	0.169
(vi) Family history of gastric cancer	3.22	3.57	5.15	0.663
CD44 polymorphism (%)				
(i) AA	12.90	17.90	2.50	0.024^*∗*^
(ii) AG	45.20	39.30	42.50	0.683
(iii) GG	41.90	42.90	55.0	0.425
CD44 protein staining (positive (*n*) (%))	-	21.40	65.0	0.036^*∗*^

Comparison between the groups was done by using ANOVA. ^*∗*^Significance is set at *p* < 0.05.

**Table 2:** The distribution of the gastric mucosal pathology related to expression of CD44 and polymorphisms.

Polymorphism of gastric mucosal pathology	Expression of CD44 protein (%)		OR (95% CI)	*p* value
	Negative	Positive		
Precancerous (*n* = 113)^a^				
(i) AA	10.71	7.14	0.68 (0.47–0.96)	0.693
(ii) AG	35.71	14.28	0.91 (0.87–1.86)	0.058
(iii) GG	28.57	-	-	-
Gastric cancer (*n* = 162)^a^				
(i) AA	-	2.56	-	-
(ii) AG	7.69	35.89	5.67 (1.54–7.23)	0.024^*∗*^
(iii) GG	10.25	43.58	8.32 (2.94–11.42)	0.016^*∗*^
Gastric cancer (*n* = 162)^b^				
(i) AG	7.69	35.89	3.28 (2.47–5.63)	0.036^*∗*^
(ii) GG	10.25	43.58	4.14 (1.84–7.32)	0.046^*∗*^

^a^Univariate Cox regression model analysis used to analyze the data. ^b^Multivariate Cox proportional hazards regression model analysis was used to analyze the data. OR, odds ratio; CI, confidence interval. ^*∗*^Significance is set at *p* < 0.05.

**Table 3:** Clinicopathological characteristics of gastric cancer associated with CD44 expression (univariate Cox regression model analysis).

Clinicopathological characteristics of gastric cancer (*n* = 62)	Expression of CD 44 protein (%)		OR (95% CI)	*p* value
	Negative	Positive		
Location of tumor (%)				
(i) Upper	5.12	10.25	0.62 (0.48–0.87)	0.724
(ii) Middle	10.25	15.38	0.87 (0.64–1.09)	0.953
(iii) Lower	7.69	58.84	4.69 (2.01–6.48)	0.014^*∗*^
Tumor size (%)				
(i) ≥70 mm	7.69	48.71	5.74 (3.18–8.29)	0.019^*∗*^
(ii) ≤70 mm	20.51	23.07	0.54 (0.42–0.76)	0.834
Histologic type (%)				
(i) Differentiated	23.07	17.94	0.73 (0.59–0.92)	0.524
(ii) Undifferentiated	5.12	53.84	8.29 (6.38–12.19)	0.014^*∗*^
Lymphatic invasion (%)				
(i) Absent	41.02	7.69	4.21 (1.29–7.74)	0.028^*∗*^
(ii) Present	12.82	38.46	3.39 (1.69–5.58)	0.032^*∗*^
Vascular invasion (%)				
(i) Absent	30.76	20.51	0.79 (0.62–1.38)	0.497
(ii) Present	25.64	23.07	0.59 (0.42–1.18)	0.326
Pathological T stage (%)				
(i) T1-T2	30.76	28.20	0.82 (0.63–1.13)	0.284
(ii) T3-T4	20.51	46.15	2.19 (1.02–6.29)	0.039^*∗*^
Pathological TNM stage (%)				
(i) I	7.69	5.12	0.92 (0.76–1.32)	0.562
(ii) II	17.97	12.82	0.72 (0.51–1.15)	0.481
(iii) III	5.12	28.02	4.49 (1.12–7.84)	0.010^*∗*^
(iv) IV	2.56	20.51	3.32 (1.45–5.890)	0.029^*∗*^
Residual tumor [*n* = 29] (%)				
(i) No	58.97	15.38	2.89 (1.07–4.69)	0.039^*∗*^
(ii) Microscopic	2.56	10.25	1.54 (1.01–4.43)	0.041^*∗*^
(iii) Gross (unresectable)	5.12	7.69	0.74 (0.53–0.91)	0.624
CEA (%)				
(i) <5.0 (ng/ml)	28.02	33.33	0.69 (0.58–1.42)	0.573
(ii) ≥5.0 (ng/ml)	17.94	20.51	0.98 (0.75–1.54)	0.782
5 years survival (available *n* = 149) (%)	55.03	14.76	2.83 (1.98–5.62)	0.028^*∗*^

Univariate Cox regression model analysis used to analyze the data. OR, odds ratio; CI, confidence interval. ^*∗*^Significance is set at *p* < 0.05.

**Table 4:** The distribution of the clinicopathological characteristics of gastric cancer related to expression of CD44 and polymorphisms (multivariate Cox proportional hazards regression model analysis).

Clinicopathological characteristics of gastric cancer (*n* = 162)	Expression of CD 44 protein (%)		OR (95% CI)	*p* value
	Negative	Positive		
Location of tumor (%)				
(i) Lower	7.69	58.84	3.82 (1.31–5.84)	0.018^*∗*^
Tumor size (%)				
(i) ≥70 mm	7.69	48.71	5.24 (3.28–8.18)	0.023^*∗*^
Histologic type (%)				
(i) Undifferentiated	5.12	53.84	4.19 (4.18–10.29)	0.037^*∗*^
Lymphatic invasion (%)				
(i) Absent	41.02	7.69	4.12 (1.08–6.34)	0.018^*∗*^
(ii) Present	12.82	38.46	1.09 (1.19–2.47)	0.061
Pathological T stage (%)				
(i) T3-T4	20.51	46.15	2.09 (1.02–3.07)	0.041^*∗*^
Pathological TNM stage (%)				
(i) III	5.12	28.02	3.46 (1.82–5.24)	0.026^*∗*^
(ii) IV	2.56	20.51	4.33 (3.47–8.89)	0.019^*∗*^
Residual tumor [*n* = 29] (%)				
(i) No	58.97	15.38	3.29 (1.87–4.39)	0.022^*∗*^
(ii) Microscopic	2.56	10.25	0.94 (1.31–3.83)	0.057
5-year survival (available *n* = 118) (%)	55.08	18.64	2.26 (2.01–4.89)	0.038^*∗*^

Multivariate Cox proportional hazards regression model analysis was used to analyze the data. OR, odds ratio; CI, confidence interval. ^*∗*^Adjustments were performed by incorporating all of the relevant factors, such as age, gender, underlying disease, and family history of gastric cancer into the analysis. ^*∗*^Significance is set at *p* < 0.05.

## References

[1] Torre L. A., Bray F., Siegel R. L., Ferlay J., Lortet-Tieulent J. (2015). Global cancer statistics, 2012. *CA: A Cancer Journal for Clinicians*.

[2] Wroblewski L. E., Peek R. M., Wilson K. T. (2010). Helicobacter pylori and gastric cancer: factors that modulate disease risk. *Clinical Microbiology Reviews*.

[3] Kusters J. G., Van Vliet A. H. M., Kuipers E. J. (2006). Pathogenesis of Helicobacter pylori infection. *Clinical Microbiology Reviews*.

[4] Talmadge J. E., Fidler I. J. (2010). AACR centennial series: the biology of cancer metastasis: historical perspective. *Cancer Research*.

[5] Hohenberger P., Gretschel S. (2003). Gastric cancer. *The Lancet*.

[6] Sampieri K., Fodde R. (2012). Cancer stem cells and metastasis. *Seminars in Cancer Biology*.

[7] Clarke M. F., Dick J. E., Dirks P. B. (2006). Cancer stem cells—perspectives on current status and future directions: AACR workshop on cancer stem cells. *Cancer Research*.

[8] Takaishi S., Okumura T., Wang T. C. (2008). Gastric cancer stem cells. *Journal of Clinical Oncology*.

[9] Takaishi S., Okumura T., Tu S. (2009). Identification of gastric cancer stem cells using the cell surface marker CD44. *Stem Cells*.

[10] Tan D., Kirley S., Li Q. (2003). Loss of Cables protein expression in human non-small cell lung cancer: a tissue microarray study. *Human Pathology*.

[11] Saikawa Y., Fukuda K., Takahashi T., Nakamura R., Takeuchi H., Kitagawa Y. (2010). Gastric carcinogenesis and the cancer stem cell hypothesis. *Gastric Cancer*.

[12] Yong C.-S., Ou Yang C.-M., Chou Y.-H., Liao C.-S., Lee C.-W., Lee C.-C. (2012). CD44/CD24 expression in recurrent gastric cancer: a retrospective analysis. *BMC Gastroenterology*.

[13] Collins A. T., Berry P. A., Hyde C., Stower M. J., Maitland N. J. (2005). Prospective identification of tumorigenic prostate cancer stem cells. *Cancer Research*.

[14] Dalerba P., Dylla S. J., Park I.-K. (2007). Phenotypic characterization of human colorectal cancer stem cells. *Proceedings of the National Acadamy of Sciences of the United States of America*.

[15] Park S. Y., Lee H. E., Li H., Shipitsin M., Gelman R., Polyak K. (2010). Heterogeneity for stem cell-related markers according to tumor subtype and histologic stage in breast cancer. *Clinical Cancer Research*.

[16] Ghaffarzadehgan K., Jafarzadeh M., Raziee H. R. (2008). Expression of cell adhesion molecule CD44 in gastric adenocarcinoma and its prognostic importance. *World Journal of Gastroenterology*.

[17] Cao L., Hu X., Zhang J., Liang P., Zhang Y. (2014). CD44(+) CD324(-) expression and prognosis in gastric cancer patients. *Journal of Surgical Oncology*.

[18] Wang W., Dong L.-P., Zhang N., Zhao C.-H. (2014). Role of cancer stem cell marker CD44 in gastric cancer: a meta-analysis. *International Journal of Clinical and Experimental Medicine*.

[19] Winder T., Ning Y., Yang D. (2011). Germline polymorphisms in genes involved in the CD44 signaling pathway are associated with clinical outcome in localized gastric adenocarcinoma. *International Journal of Cancer*.

[20] Zhou J., Nagarkatti P. S., Zhong Y., Zhang J., Nagarkatti M. (2011). Implications of single nucleotide polymorphisms in CD44 exon 2 for risk of breast cancer. *European Journal of Cancer Prevention*.

[21] Chou Y.-E., Hsieh M.-J., Chiou H.-L., Lee H.-L., Yang S.-F., Chen T.-Y. (2014). CD44 gene polymorphisms on hepatocellular carcinoma susceptibility and clinicopathologic features. *BioMed Research International*.

[22] Jiang L., Deng J., Zhu X. (2012). CD44 rs13347 C>T polymorphism predicts breast cancer risk and prognosis in Chinese populations. *Breast Cancer Research*.

[23] Suenaga M., Yamada S., Fuchs B. C. (2015). CD44 single nucleotide polymorphism and isoform switching may predict gastric cancer recurrence. *Journal of Surgical Oncology*.

[24] Wu X.-M., Yang H.-G., Zheng B.-A., Cao H.-F., Hu Z.-M., Wu W.-D. (2015). Functional genetic variations at the microrna binding-site in the CD44 gene are associated with risk of colorectal cancer in Chinese populations. *PLoS ONE*.

[25] Suga T., Ishikawa A., Kohda M. (2007). Haplotype-based analysis of genes associated with risk of adverse skin reactions after radiotherapy in breast cancer patients. *International Journal of Radiation Oncology, Biology, Physics*.

[26] Tongtawee T., Dechsukhum C., Leeanansaksiri W. (2015). Improved detection of helicobacter pylori infection and premalignant gastric mucosa using "site specific biopsy": a randomized control clinical trial. *Asian Pacific Journal of Cancer Prevention*.

[27] Nguyen L. V., Vanner R., Dirks P., Eaves C. J. (2012). Cancer stem cells: an evolving concept. *Nature Reviews Cancer*.

[28] Bomken S., Fišer K., Heidenreich O., Vormoor J. (2010). Understanding the cancer stem cell. *British Journal of Cancer*.

[29] Gerger A., Zhang W., Yang D. (2011). Common cancer stem cell gene variants predict colon cancer recurrence. *Clinical Cancer Research*.

[30] Huh J. W., Kim H. R., Kim Y. J. (2009). Expression of standard CD44 in human colorectal carcinoma: association with prognosis. *Pathology International*.

[31] Dammrich J., Vollmers H. P., Heider K.-H., Muller-Hermelink H.-K. (1995). Importance of different CD44v6 expression in human gastric intestinal and diffuse type cancers for metastatic lymphogenic spreading. *Journal of Molecular Medicine*.

[32] Vazquez A., Grochola L. F., Bond E. E. (2010). Chemosensitivity profiles identify polymorphisms in the p53 network genes 14-3-3*τ* and CD44 that affect sarcoma incidence and survival. *Cancer Research*.

